# Morphological Characterization of Merino Sheep in Different Agro‐Ecological Zones of Lesotho

**DOI:** 10.1155/tswj/9028576

**Published:** 2026-01-26

**Authors:** Motlalepula George, Morai Johannes Moiloa, Ouko William Odenya, Puleng Matebesi-Ranthimo, Setsomi Molapo, Manyeoe Khatite

**Affiliations:** ^1^ Department of Animal Science, The National University of Lesotho, Maseru, Lesotho

## Abstract

The Lesotho Merino sheep is a native Merino strain formed from the indigenous fat‐tailed sheep through crossbreeding over many generations. This study is aimed at phenotypically characterizing Merino sheep locally bred in four agro‐ecological zones of Lesotho, facilitating easy selection based on morphological traits. Body weight (BW), body length (BL), withers height (WH), rump height (RH), chest girth (CG), rump length (RL) and rump width (RW) were measured in 2515 mature shorn Merino ewes from four agro‐ecological zones: mountains (*n* = 1554), the Senqu River Valley (*n* = 350), lowlands (*n* = 395) and foothills (*n* = 216). A multivariate discriminant analysis procedure identified and quantified the traits that differentiate the Merino sheep across these agro‐ecological zones. The structure matrix indicated that RL had the highest loading (0.82) in Function 1, whereas WH (0.6) and head width (0.36) exhibited the highest loadings in Functions 2 and 3, respectively. The standardized canonical discriminant coefficients showed that RL (1.02) and RW (0.60) were the highest in Function 1, whereas CG (0.65) and RH (0.41) were in Function 2, and WH (1.61) and RW (0.41) were in Function 3. The Mahalanobis distance was highest between the lowlands and the Senqu River Valley (3.46) and lowest between the mountains and foothills (0.61). Principal component analysis (PCA) extracted three components per agro‐ecological zone. Morphological traits differentiate Merino sheep across the agro‐ecological zones, suggesting the presence of two strains: one suited for the mountains, the Senqu River Valley, and foothills and another suited for the lowlands.

## 1. Introduction

The Lesotho Merino sheep is a local Merino strain developed from the indigenous fat‐tailed sheep through crossbreeding over many generations since the 1800s [1]. Immigrant farmworkers led the introduction of this breed from the South African Merino population, which was selected primarily for producing medium wool grades [1]. According to the United Nations COMTRADE database on international trade, Lesotho′s wool and mohair export sector has experienced notable fluctuations from 2020 to 2023, reflecting broader economic challenges and sector‐specific dynamics [2]. The returns significantly encouraged most urban and rural communities to own sheep. The mean wool production is 2.74 kg/head/year, which, when multiplied by the population, equals 6515 tons of wool annually [3].

Currently, there are 2,377,809 heads of sheep [4] reared under the diverse environmental landscape of the country. This heterogeneous production environment has an unintentional impact on their performance, emphasising the importance of characterizing these genetic resources for a proper long‐term management plan. Farmers prioritize males over females in the large production system of communally managed rangelands. Females are rarely culled or selected; therefore, the favourable effects of direct selection are limited. Environmental factors are assumed to cause a high rate of mortality. Although sheep are generally raised for wool, growth is equally important because it impacts survival and marketability, as sheep are also sold for mutton. The emphasis on selecting males in communal rangeland systems is closely tied to morphological characterization, as observable traits that guide farmers in making breeding decisions that are crucial for the productivity and sustainability of their livestock.

The relationship between animal genetics and geographic locations and/or climatic variables is thought to be indicative of adaptation signatures, providing information on the environmental forces influencing the genome [5]. Therefore, understanding the genetic heterogeneity among breed populations is essential for effective long‐term management [6]. Measurements of various body conformations are valuable in judging the quantitative characteristics of animals and are also helpful in developing suitable selection criteria [7]. Characterizing animal diversity is essential to meeting future needs in Lesotho.

The first step in the sustainable use of livestock′s animal genetic resources is characterizing them [8] to get the body conformation concept. Body measurements have been used to identify an individual′s breed, origin, relationship, size and shape [9]. They have also been used to characterize sheep and goats [9]. Planning improvements, sustainable use, conservation tactics and breeding plans for a breed all depend on the morphometric classification of livestock [10].

Discriminant function analysis is used to predict the accuracy of a classification system or predictor variables [11]. On the other hand, principal component analysis (PCA) reduces an original group of variables into another smaller group called principal components (PCs), which are the linear combination of original variables [12, 13] to account for the maximum portion of the variance present in the original set of variables with a minimum number of composite variables.

This study is aimed at describing and quantifying phenotypic heterogeneity among Lesotho Merino sheep populations across various agro‐ecological zones using morphometric parameters.

## 2. Materials and Methods

Despite the absence of a research ethics committee in the country, the Department of Animal Science at the National University of Lesotho ensured that the study was conducted with standards that did not violate recommended animal welfare practices. The study was conducted across five districts representing four agro‐ecological zones of Lesotho: mountains (Mokhotlong, parts of Quthing and parts of Qacha′s Nek), Senqu River Valley (parts of Quthing and parts of Qacha′s Nek), foothills (parts of Mafeteng and parts of Berea) and lowlands (parts of Mafeteng and parts of Berea). The country features a temperate climate, characterized by hot summers and cold winters.

**Figure 1:**
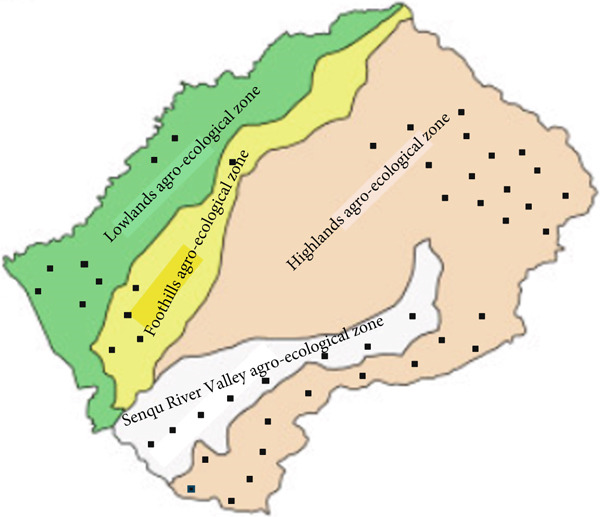
Map showing agro‐ecological zones of Lesotho and sampled shearing sheds (indicated by black squares).

Figure 1 shows the position of the sampled shearing shed in different agro‐ecological zones. The mountains′ agro‐ecological zone constitutes the largest ecological area and serves as the primary grazing region. It is located between 2130 and 3480 m above sea level (m.a.s.l.), covering approximately 18,047 km^2^ and representing 59% of the total land area of 30,355 km^2^ [14]. The mean annual temperature varies from 7°C to 20°C [15]. In winter, temperatures can drop to as low as −20°C [14]. This zone features grasslands with abundant functional groups, including C3 and C4 grasses, along with encroaching unpalatable dwarf karroid shrubs confined to low, warm and dry slopes [16]. The lowlands agro‐ecological zone covers the western part of the country and spans about 5,200 km^2^, accounting for 17% of the total surface area [14]. It is situated between 1400 and 1,800 m a.s.l [14]. The mean annual temperature ranges from 15.2°C to 32°C [15]. Winters can be chilly, with temperatures dropping as low as −7°C [15]. Poor soils and low rainfall are characteristics of this zone. The foothills agro‐ecological zone lies between the lowlands and the mountains, situated at elevations ranging from 1,800 to 2,000 m.a.s.l. [14]. It accounts for an estimated 15% of the total land area and comprises fertile soils suitable for high agricultural productivity. The Senqu River Valley, situated between 1388 and 2000 m.a.s.l., occupies 9% of the land area and is the driest region, as it is located in the rain shadow of the Maluti and Drakensberg mountain ranges [14].

**Figure 2:**
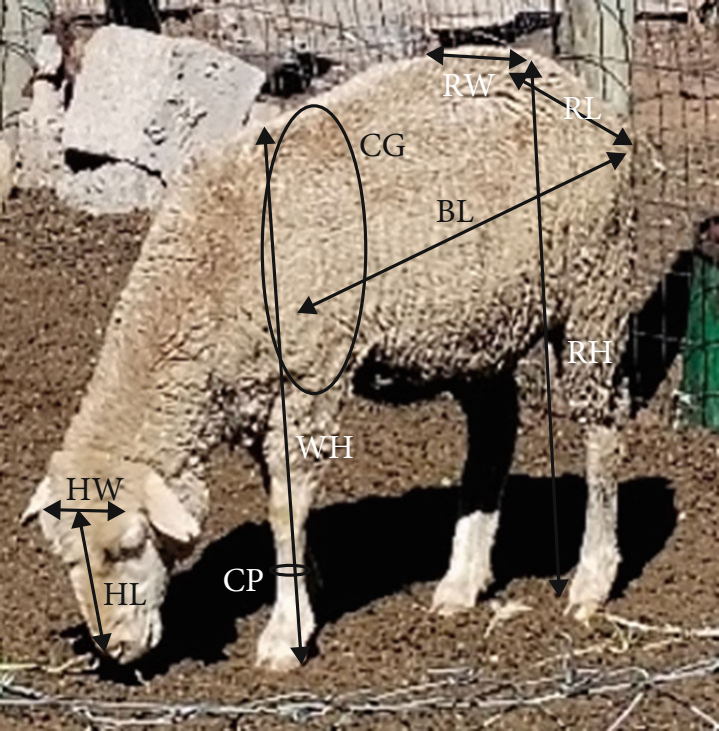
Some of the measured morphological traits: HW, head width; HL, head length; CP, cannon perimeter; WH, withers height; CG, chest girth; BL, body length; RW, rump width; RL, rump length).

The standard breed descriptor list for sheep developed by Food and Agriculture Organisation [6] was closely followed in selecting quantitative traits (body measurement) of body length (BL), head length (HL), head width (HW), withers height (WH), rump height (RH), chest girth (CG), chest depth (CD), rump length (RL), rump width (RW), ischium width (ISW) and cannon perimeter (CP). Measurements were taken using a flexible measuring tape, a steel tape and a ruler. The morphostructural traits were measured and recorded from 2515 shorn mature ewes from 48 shearing sheds and 145 farmers (15–30 animals per farmer). Figure 2 illustrates the position where the measurements were taken on the sheep. Body weights were taken from 2102 ewes using a 180 kg capacity bathroom scale, whereby the researcher carried an animal and stood on the scale. The researcher first weighed himself before carrying the animal for the combined weight. The weight of the animal was obtained by calculating the difference between the combined weight of the animal and the researcher and the weight of the researcher alone. The missing body weight data of 413 animals was caused by the malfunctioning of the weighing scales while data was collected in remote areas where the scales could not be replaced immediately.

The collected data were subjected to descriptive statistical analysis using the IBM SPSS (Version 27) statistical software. Multivariate normality was assessed using Mardia′s test; both skewness and kurtosis were significant (*p* < 0.01). The homogeneity of variance–covariance was tested using the Levene and Box′s M tests; the variance–covariance structure within each agro‐ecological zone was similar. The traits were subjected to stepwise discriminant analysis (SDA) to determine the ones that contributed to discrimination between the four agro‐ecological zones. Discriminant analysis offers superior capabilities for classifying individuals into predefined groups based on multiple interrelated traits. Its multivariate approach, consideration of covariance and classification power make it a preferred method in the morphological characterization of sheep breeds. Thus, accordingly, the agro‐ecological zone was the nominal grouping variable and the classification variable, whereas the morphometric traits were the predictor variables. The analysis created a discriminant function, which was a linear combination of the weightings and scores on those variables. The maximum number of functions was the number of groups minus one. The discriminant model has the following mathematical form for each function: *Z*_*j* *k*_ = a + *W*_1_ *X*_1*k*_ + W_2_X_2k_ + ⋯+W_n_X_nk_, where *Z*_*j* *k*_, discriminant score of discriminant function *j* for object *k*;*a*,ntercept; *W*_*i*_ , discriminant coefficient for the independent variable *i*; *X*_*j*_, independent variable *i* for object *k*.

The percentage of misclassified animals indicated the degree of admixture between the agro‐ecological zones. Canonical discriminant analysis was performed to obtain the Mahalanobis distance of agro‐ecological zones on all phenotypic traits.

Means, standard deviation and correlation coefficients were computed. From the correlation matrix, data were generated for the PC factor analysis using the factor programme in SPSS. The PC analysis was verified for adequate determinant factor sample adequacy using the Kaiser–Meyer–Olkin (KMO) test and sphericity using Bartlett′s test.

## 3. Results and Discussion

Of the four agro‐ecological zones, sheep from the lowlands had the highest average for most morphometric traits (Table 1), indicating that the lowland environment allowed the sheep to perform at their best for the measured traits compared with sheep in other agro‐ecological zones. The large variation shown by higher standard deviations in some measurements was a result of the absence of selection or the fact that some body parts are affected differently by the environment than others. Overall, there were significant differences in almost all body measurements across the agro‐ecological zones, suggesting that animals adapted differently to the agro‐ecological zones.

**Table 1:** Means ± SD of body measurements for different agro‐ecological zones.

**Variable**	**Mountains**	**Senqu River Valley**	**Lowlands**	**Foothills**
Head length	19.87^c^ ± 1.54	19.88^c^ ± 1.28	20.70^a^ ± 1.34	20.29^b^ ± 1.31
Head width	12.83^b^ ± 0.86	12.84^b^ ± 0.77	13.02^a^ ± 0.81	12.87^b^ ± 0.75
Withers height	64.19^b^ ± 4.44	63.69^c^ ± 3.85	66.21^a^ ± 3.85	63.80^c^ ± 3.97
Rump height	65.03^b^ ± 4.44	65.01^b^ ± 3.79	67.16^a^ ± 3.71	64.47^c^ ± 3.71
Rump length	23.18^c^ ± 1.96	23.94^c^ ± 1.79	26.36^a^ ± 1.52	25.04^b^ ± 1.99
Rump width	16.77^d^ ± 1.66	17.17^c^ ± 1.78	19.18^a^ ± 1.30	17.87^b^ ± 1.53
Ischium width	18.75^d^ ± 1.57	19.14^c^ ± 1.44	19.93^a^ ± 1.43	19.47^b^ ± 1.55
Chest girth	79.99^c^ ± 6.09	80.87^b^ ± 6.36	85.65^a^ ± 4.75	81.14^b^ ± 5.99
Body length	75.96^c^ ± 5.98	76.40^c^ ± 5.42	79.86^a^ ± 4.85	77.21^b^ ± 5.23
Cannon perimeter	7.7^b^ ± 0.7	7.7^b^ ± 0.6	8.0^a^ ± 0.4	7.8^b^ ± 0.5
Body weight	39.46^c^ ± 7.25	39.12^c^ ± 5.99	45.57^a^ ± 7.17	41.11^b^ ± 6.27

*Note:* Means with different superscripts in a row are significantly different.

The lowland animals had morphological trait measurements that suggested superior skeletal and muscle development, indicating that the environment in this zone is suitable for meat production as a secondary trait, wool production being the primary trait. The sheep from the lowlands had the highest mean body weight (45.6), followed by the foothills (41.1), then the mountains (39.5) and lastly the Senqu River Valley (39.1) (Table 1). However, a high standard deviation indicates a wide range of trait values, suggesting considerable diversity within that population. This could be due to genetic factors or varying farmer management practices. The findings were at variance with those reported by [17, 18], who found that district and agro‐ecological zones do not affect body weight.

The Wilks′ Lambda results yielded *p* values of zero (0.000), indicating that the model is an effective fit for the data (Table 2). The Wilks′ Lambda, or the unexplained variance, tests the hypothesis that the means of the four agro‐ecological zones are equal. The null hypothesis is rejected; thus, the means of the agro‐ecological zones differ. This suggests that the predictors (i.e., morphological traits) possess a discriminatory ability. The Box′s M test for the equality of covariance matrices was significant (< 0.001) and showed that these matrices were not equal.

**Table 2:** Wilks′ Lambda results.

**Test of functions**	**Wilks′ lambda**	**Chi-square**	**Df**	**Sig**
1 through 3	0.528	1363.126	21	0
2 through 3	0.937	138.196	12	0
3	0.992	17.079	5	0.004

**Eigenvalue, percentage variance, cumulative and canonical correlation (r)**				
**Function**	**Eigenvalue**	**Variance %**	**Cumulative %**	**Canonical r**
1	0.774^a^	92.1	92.1	0.661
2	0.058^a^	6.9	99	0.235
3	0.008^a^	1	100	0.089

^a^First 3 canonical discriminant functions were used in the analysis.

The percentage share of variance (90.5) in Function 1 was greater than 7.8% and 1.8% in Functions 2 and 3, respectively. The canonical correlation in Function 1 was also higher (0.662) than the canonical correlations in Functions 2 and 3 (Table 2). There was statistical significance (*p* = 0.000) in Function 1, with a small lambda of 0.519, accounting for 90.5% of the variance. Although the other two functions were significant (*p* = 0.000), their effects were minimal. Therefore, they did not contribute significantly to the discrimination process between the groups compared with that of the first function.

The canonical correlation statistic measures the association between the discriminant scores and the grouping variable, agro‐ecological zone. In Function 1, the correlation was 0.662, which is reasonably high, indicating that the model possesses predictive ability. The Wilks′ Lambda results were similar to those reported by [19] for indigenous sheep in the western part of Ethiopia. The first two canonical variables accounted for 98.2% of the total variation, a figure higher than 80.2% obtained by [20] in Saudi sheep breeds. The larger share of variance (90.5%) in the grouping variable, agro‐ecological zone, was explained with only one function. The results were consistent with those reported by [21, 22].

The standardized canonical discriminant coefficients, arranged in hierarchical order by the importance of traits within functions, show that, in Function 1, the strongest positive contributors: RL (1.021) and RW (0.603)—sheep with longer, wider rumps score higher on this function. Strongest negative contributors are ISW (−0.326), RH (−0.273) and HW (−0.215). This function separates groups with broader, longer rear ends from those with narrower, shorter ones. Other measurements (e.g., WH and CG) have minimal impact. In Function 2, the strongest positive contributors: CG (0.654), RH (0.413), and WH (0.412)—sheep with larger chests, taller rumps, and higher withers score higher. Strongest negative contributors: ISW (−0.746) and RL (−0.547). It discriminates taller, broader‐chested sheep from shorter, narrower ones. RW also contributes positively but less strongly.

In Function 3, the strongest positive contributors are WH (1.609) and RW (0.411)—higher withers and wider rumps increase the score. Strongest negative contributors are RH (−1.684). This separates sheep with elevated withers (front height) relative to RH, versus those with more level or lower rump profiles. Other measurements have smaller effects (Table 3). Although the same traits were important in the three functions, the magnitude and sign of the coefficients were different in some functions. This indicates the intervariation of these traits that differentiate the Merino sheep among the agro‐ecological zones. RL, RW, CG and WH are traits that can be selected for improving meat and wool production efficiency. The three functions in this study were equal to those stated by [23] for goats, and they exceeded the two reported by [24] for Serbian autochthonous goats.

**Table 3:** Standardized canonical discriminant coefficients.

**Trait**	**Function**		
	**1**	**2**	**3**
Head width	−0.215	−0.262	0.309
Withers height	−0.195	0.412	1.609
Rump height	−0.273	0.413	−1.684
Rump length	1.021	−0.547	−0.029
Rump width	0.603	0.492	0.411
Ischium width	−0.326	−0.746	−0.29
Chest girth	−0.007	0.654	−0.284

Based on the standardized canonical discriminant coefficients, the models for the prediction equation, which could be used for classifying the new cases, are as follows:

DF_1_ = −0.215 HW–0.195 WH–0.273 RH + 1.021 RL + 0.603 RW–0.326 ISW–0.007CG

DF_2_ = −0.262 HW + 0.412 WH + 0.413 RH–0.547 RL + 0.492 RW–0.746 ISW + 0.654 CG 

DF_3_ = 0.309 HW + 1.609 WH–1.684RH–0.029RL + 0.411RW–0.29ISW–0.28CG, where DF_1, 2 and 3_, discriminant functions; HW, head width; WH, withers height; RH, rump height; R, rump length; R, rump width; ISW, ischium width and CG, chest girth.

For classification, the group cases that were correctly classified in the mountains were 93%. Misclassified percentages were 0.7%, 5% and 1.6% in the Senqu River Valley, lowlands and foothills agro‐ecological zones, respectively (shaded and above diagonal part of Table 4). The group cases in the mountains were classified correctly, with smaller percentages misclassified in the Senqu River Valley, lowlands and foothills agro‐ecological zones, respectively.

**Table 4:** Mahalanobis distance between and classification results of agro‐ecological zones.

**Agro-ecological zone**	**Mountains**	**Senqu River Valley**	**Lowlands**	**Foothills**
Mountains	93	0.7/79	5/21	1.6/52
Senqu River Valley	1.018	1.7	15/0.5	3.7/2.3
Lowlands	2.437	3.455	74	4.6/34
Foothills	0.607	1.625	1.83	12

*Note:* A total of 70.4% of the original grouped cases were correctly classified.

A larger percentage (74%) of sheep from the lowlands were correctly classified, with 21%, 0.5%, and 4.6% being misclassified into mountains, Senqu River Valley and foothills agro‐ecological zones, respectively. In the foothills, 12% of sheep were correctly classified. Sheep misclassified into the mountains, Senqu River Valley and lowlands zones were 52%, 2.3% and 34%, respectively. The low misclassification within the mountains may be an indication of the uniformity of the Merino breed in the agro‐ecological zones. The overall percentage of the original cases that were correctly classified was 70.4%. This was higher than the 65% reported by [19].

The unshaded (below diagonal) part of Table 4 illustrates the Mahalanobis distances from each agro‐ecological zone. The greatest distance was recorded between the Senqu River Valley and the lowlands agro‐ecological zones (3.455), followed by the mountains and lowlands agro‐ecological zones (2.437). The smallest distance was noted between the mountains and foothills agro‐ecological zones (0.607). A larger distance between the Senqu River Valley and the lowlands indicates greater genetic divergence, suggesting that the populations have adapted to different environments or have been subjected to varying selective pressures. The low distance between the mountains and the foothills may be attributed to the fact that most of the foothills′ animals spend much or all of their time in the mountains′ rangelands due to the transhumance system and the geographical proximity of the two zones. These phenomena result in some gene exchange between animals of the two agro‐ecological zones.

The large differences between agro‐ecological zones show that there are likely to be differences among Merino breed strains. Thus, it is plausible that there are two strains of the Merino breed, one for the mountains, foothills and Senqu Valley and the other for the lowlands. These figures were higher than the 2.27 reported by [19]. The distance between the mountains and foothills agro‐ecological zones (0.607) is lower than 0.649 reported by [19].

Correlation coefficients between traits for the mountains and Senqu River Valley agro‐ecological zones are shown in Table 5. CG had the highest correlation with body weight at 0.77 for mountains, 0.71 for the Senqu River Valley zone, 0.539 in the lowlands and 0.703 in the foothills. The mountains zone correlations ranged from 0.305 (CD and CP) to 0.867 (withers and RHs). This indicated that unrelated sets of genes controlled both CD and CP. On the other hand, WH and RH are likely controlled by many of the same genes, located on the same locus or highly related genes. Sheep with larger CG typically have better lung capacity and overall vitality, which can influence their performance in various production systems. Taller sheep tend to weigh more, which can indicate better growth potential and overall health.

**Table 5:** Pearson′s correlation coefficients between the traits for the mountains below the diagonal and the Senqu River Valley agro‐ecological zone above the diagonal.

	**BW**	**HL**	**HW**	**WH**	**RH**	**CD**	**RL**	**RW**	**ISW**	**CG**	**CP**	**BL**
BW	1.00	0.554 ^∗^	0.388 ^∗^	0.523 ^∗^	0.533 ^∗^	0.428 ^∗^	0.520 ^∗^	0.555 ^∗^	0.566 ^∗^	0.711 ^∗^	0.258 ^∗^	0.690 ^∗^
HL	0.579 ^∗^	1.00	0.377 ^∗^	0.561 ^∗^	0.553 ^∗^	0.463 ^∗^	0.558 ^∗^	0.433 ^∗^	0.565 ^∗^	0.640 ^∗^	0.377 ^∗^	0.577 ^∗^
HW	0.483 ^∗^	0.510 ^∗^	1.00	0.369 ^∗^	0.394 ^∗^	0.206 ^∗^	0.361 ^∗^	0.427 ^∗^	0.420 ^∗^	0.400 ^∗^	0.224 ^∗^	0.426 ^∗^
WH	0.586 ^∗^	0.615 ^∗^	0.511 ^∗^	1.00	0.822 ^∗^	0.481 ^∗^	0.534 ^∗^	0.466 ^∗^	0.557 ^∗^	0.539 ^∗^	0.338 ^∗^	0.580 ^∗^
RH	0.582 ^∗^	0.636 ^∗^	0.527 ^∗^	0.867 ^∗^	1.00	0.435 ^∗^	0.534 ^∗^	0.456 ^∗^	0.534 ^∗^	0.572 ^∗^	0.344 ^∗^	0.605 ^∗^
CD	0.498 ^∗^	0.518 ^∗^	0.434 ^∗^	0.624 ^∗^	0.594 ^∗^	1.00	0.433 ^∗^	0.399 ^∗^	0.429 ^∗^	0.558 ^∗^	0.228 ^∗^	0.457 ^∗^
RL	0.573 ^∗^	0.613 ^∗^	0.453 ^∗^	0.567 ^∗^	0.596 ^∗^	0.447 ^∗^	1.00	0.628 ^∗^	0.662 ^∗^	0.685 ^∗^	0.418 ^∗^	0.585 ^∗^
RW	0.638 ^∗^	0.586 ^∗^	0.506 ^∗^	0.623 ^∗^	0.634 ^∗^	0.575 ^∗^	0.608 ^∗^	1.00	0.648 ^∗^	0.596 ^∗^	0.212 ^∗^	0.538 ^∗^
ISW	0.639 ^∗^	0.632 ^∗^	0.505 ^∗^	0.608 ^∗^	0.632 ^∗^	0.530 ^∗^	0.705 ^∗^	0.751 ^∗^	1.00	0.693 ^∗^	0.345 ^∗^	0.625 ^∗^
CG	0.771 ^∗^	0.608 ^∗^	0.520 ^∗^	0.627 ^∗^	0.638 ^∗^	0.573 ^∗^	0.681 ^∗^	0.705 ^∗^	0.716 ^∗^	1.00	0.425 ^∗^	0.707 ^∗^
CP	0.426 ^∗^	0.324 ^∗^	0.347 ^∗^	0.379 ^∗^	0.383 ^∗^	0.305 ^∗^	0.366 ^∗^	0.367 ^∗^	0.367 ^∗^	0.504 ^∗^	1.00	0.345 ^∗^
BL	0.643 ^∗^	0.597 ^∗^	0.474 ^∗^	0.632 ^∗^	0.640 ^∗^	0.502 ^∗^	0.673 ^∗^	0.623 ^∗^	0.658 ^∗^	0.687 ^∗^	0.434 ^∗^	1.00

Abbreviations: BL, body length; BW, body weight; CD, chest depth; CG, chest girth; CP, cannon circumference; HL, head length; HW, head width; ISW, ischium width; RH, rump height; RL, rump length; RW, rump width; WH, withers height.

^∗^ *p* < 0.01.

The lowland correlations ranged from 0.011 (HW and CP) to 0.857 (WH and RH). The variables were mostly moderately correlated with the determinant of 0.008. The foothill correlations ranged from 0.112 (CP and CD) to 0.837 (RH and WH), with the determinant being 0.002 (Table 6). The determinant was 0.003, indicating the existence of moderate multicollinearity of the variables. The highest correlation between CG and body weight was also reported by [25] for indigenous sheep types in the Bale zone.

**Table 6:** Pearson′s correlation coefficients between the traits for the lowlands below the diagonal and the foothills agro‐ecological zone above the diagonal.

	**BW**	**HL**	**HW**	**WH**	**RH**	**CD**	**RL**	**RW**	**ISW**	**CG**	**CP**	**BL**
BW	1.00	0.579 ^∗^	0.441 ^∗^	0.547 ^∗^	0.657 ^∗^	0.376 ^∗^	0.625 ^∗^	0.524 ^∗^	0.440 ^∗^	0.703 ^∗^	0.358 ^∗^	0.635 ^∗^
HL	0.490 ^∗^	1.00	0.429 ^∗^	0.535 ^∗^	0.575 ^∗^	0.382 ^∗^	0.558 ^∗^	0.407 ^∗^	0.407 ^∗^	0.581 ^∗^	0.301 ^∗^	0.493 ^∗^
HW	0.350 ^∗^	0.382 ^∗^	1.00	0.424 ^∗^	0.390 ^∗^	0.379 ^∗^	0.376 ^∗^	0.248 ^∗^	0.231 ^∗^	0.373 ^∗^	0.255 ^∗^	0.421 ^∗^
WH	0.386 ^∗^	0.460 ^∗^	0.203 ^∗^	1.00	0.837 ^∗^	0.557 ^∗^	0.576 ^∗^	0.389 ^∗^	0.283 ^∗^	0.493 ^∗^	0.238 ^∗^	0.583 ^∗^
RH	0.433 ^∗^	0.487 ^∗^	0.147 ^∗^	0.857 ^∗^	1.00	0.441 ^∗^	0.621 ^∗^	0.461 ^∗^	0.399 ^∗^	0.590 ^∗^	0.283 ^∗^	0.615 ^∗^
CD	0.310 ^∗^	0.251 ^∗^	0.146 ^∗^	0.472 ^∗^	0.469 ^∗^	1.00	0.534 ^∗^	0.342 ^∗^	0.156 ^∗^	0.459 ^∗^	0.112	0.555 ^∗^
RL	0.455 ^∗^	0.339 ^∗^	0.252 ^∗^	0.450 ^∗^	0.471 ^∗^	0.275 ^∗^	1.00	0.568 ^∗^	0.386 ^∗^	0.618 ^∗^	0.278 ^∗^	0.658 ^∗^
W	0.386 ^∗^	0.204 ^∗^	0.109 ^∗^	0.322 ^∗^	0.348 ^∗^	0.302 ^∗^	0.425 ^∗^	1.00	0.629 ^∗^	0.582 ^∗^	0.295 ^∗^	0.469 ^∗^
ISW	0.500 ^∗^	0.356 ^∗^	0.283 ^∗^	0.314 ^∗^	0.334 ^∗^	0.208 ^∗^	0.478 ^∗^	0.495 ^∗^	1.00	0.468 ^∗^	0.381 ^∗^	0.313 ^∗^
CG	0.539 ^∗^	0.405 ^∗^	0.086	0.422 ^∗^	0.473 ^∗^	0.420 ^∗^	0.452 ^∗^	0.461 ^∗^	0.423 ^∗^	1.00	0.301 ^∗^	0.597 ^∗^
P	0.117	0.265 ^∗^	0.011	0.133 ^∗^	0.217 ^∗^	0.137 ^∗^	0.093	0.137 ^∗^	0.095	0.190 ^∗^	1.00	0.289 ^∗^
BL	0.501 ^∗^	0.434 ^∗^	0.346 ^∗^	0.478 ^∗^	0.480 ^∗^	0.256 ^∗^	0.436 ^∗^	0.283 ^∗^	0.438 ^∗^	0.402 ^∗^	0.103 ^∗^	1.00

Abbreviations: BL, body length; BW, body weight; CD, chest depth; CG, chest girth; CP, cannon circumference; HL, head length; HW, head width; ISW, ischium width; RH, rump height; RL, rump length; RW, rump width; WH, withers height.

^∗^ *p* < 0.01.

The KMO values were 0.94, 0.90, 0.85 and 0.88 for the mountains, Senqu River Valley, lowlands and foothills, respectively (Table 7). A KMO value within the range of 0.6–0.7 is typically a good measure of factor suitability [26]. KMO results generally reflect the factor suitability. A high KMO value and a significant Bartlett′s test indicate that the data are suitable for further analysis to explore underlying factors that may influence sheep traits and performance in different environmental contexts.

**Table 7:** Kaiser–Meyer–Olkin (KMO) and Bartlett′s test for the agro‐ecological zones.

**KMO and Bartlett′s test**		**Mountains**	**Senqu River Valley**	**Lowlands**	**Foothills**
KMO Measure of sampling adequacy		0.939	0.899	0.852	0.884
Approx. chi‐square		10025.527	1847.795	1728.542	1262.394
Bartlett′s test of sphericity	Df	66	66	66	66
	Sig.	0.00	0.00	0.00	0.00

Further significance of the correlation matrix with Bartlett′s test for sphericity (*p* < 0.000) indicated that the correlation matrices for agro‐ecological zones were not orthogonal, thus rejecting the null hypotheses of the existence of identity matrices (which consist of all zeros except the 1′s along the diagonal). This indicates that it is improbable to have obtained the observed correlation matrices from populations with zero correlations. The KMO values in this study were higher than the 0.60 reported for Assam hill goat in eastern Himalayan India [27].

Eigenvalues (quality scores) are the most commonly used index for determining the number of components to take from a PCA. Since the eigenvalue represents the amount of standardized variance in the variables accounted for by a factor, it should represent at least as much variance as contained in a single variable. Thus, an eigenvalue of at least one determines the number of components to choose. Only components with high eigenvalues (at least 1) will likely represent a real underlying component.

Eigenvalues and factor loading after varimax rotation and communality results for the mountains and Senqu River Valley zones are shown in Table 8. For the mountains, PC1 explained 33.50% of the variance ,whereas PC2 and PC3 explained 27.24% and 11.57% of the variance, respectively. In the Senqu River Valley, PC1, PC2 and PC3 explained 30.26%, 24.01% and 9.89% of the variance, respectively. In both the mountains and Senqu River Valley rotated components, ISW, RL, RW and CG had high loadings in the first component. WH, RH and CD had high loadings on the second component, whereas CP had high loadings on the third component. The first PC (shape and general body size) represented a weighted average of the eight traits. The second PC had its loading for meat traits. The third component loads for stability and traction traits. The PCA of all morphometric parameters indicated that the three components accounted for 72% and 64% of the cumulative variance in the mountains and Senqu River Valley agro‐ecological zones, respectively.

**Table 8:** Eigenvalues and factor loading after varimax rotation, variation and cumulative percentage for principal components.

**Traits**	**Mountains**			**Senqu River valley**			**Lowlands**			**Foothills**		
	**PC1**	**PC2**	**PC3**	**PC1**	**PC2**	**PC3**	**PC1**	**PC2**	**PC3**	**PC1**	**PC2**	**PC3**
Body weight	0.68			0.67	0.51		0.57		0.50	0.59	0.49	
Head length	0.54	0.58			0.55			0.46	0.63	0.45		0.49
Head width		0.60		0.52					0.85	0.48		0.56
Withers height		0.81			0.83			0.80		0.78		
Rump height	0.40	0.79			0.80			0.82		0.70		
Chest depth		0.72			0.62			0.62		0.79		
Rump length	0.79			0.69			0.63			0.66	0.49	
Rump width	0.74			0.83			0.81				0.84	
Ischium width	0.81			0.75			0.73				0.80	
Chest girth	0.71			0.72			0.64	0.41		0.50	060	
Cannon perimeter			0.93			0.85		0.45				0.77
Body length	0.67			0.58	0.49				0.58	0.71		
Eigenvalues	4.02	3.27	1.39	3.63	2.88	1.19	2.73	2.49	2.00	3.75	2.55	1.68
% Variation	33.5	27.2	11.6	30.3	24.0	9.89	22.7	20.7	16.7	31.2	21.3	14.0
Cumulative %	72.31			64.16			60.13			66.47		

Abbreviations: PC1, Principal Component 1; PC2, Principal Component 2; PC3, Principal Component 3.

In the lowlands, PC1, PC2 and PC3 explained 22.7%, 20.7% and 16.7% of the variance, respectively, whereas in the foothills, PC1, PC2 and PC3 explained 31.2%, 21.3% and 14.0% of the variance, respectively (Table 8). RL, RW, ISW, CG and body weight load for the first component in the lowlands, which may be attributed to meat traits. WH, RH and CD load for the second component (body size). BL, HW and length are loaded for the third component (body shape). In the foothills, BL, RL, CD, RH, WH and body weight load for the first component (body size). CG, ISW and RW were loaded for the second component (meat traits). CP was loaded for the third component (traction traits). The PCA of all morphometric parameters indicated that the three components accounted for 60% and 66% of the cumulative variance in the lowlands and foothills agro‐ecological zones, respectively.

The mountains′ results were similar to those reported by [27]; who stated that the first PC (PC1) was characterized by BL, RL, heart girth (HG), paunch girth (PG) and sacral pelvic width (SPW) and contributed the largest portion of the total variance (40.37%), which might be considered as a body size factor. The results of component extraction were consistent with the findings of [28], which indicated that the first factor explained the largest percentage of the total variance. The number of components was equal to those reported by [29] for Alpine and Saana but less than the four reported by [30] for Katjang goats in Indonesia and [31] for creole sheep.

## 4. Conclusion

The SDA produced three prediction equations that could be used to classify the additional examples. Function 1 contributes the most to group separation, followed by 2 and 3, based on the stepwise selection. Together, these functions classify sheep by combining rump dimensions (Function 1), height/girth (Function 2) and vertical proportions (Function 3). The results demonstrate that there was some gene‐environment interaction; therefore, similar Merino sheep genotypes can be employed in three agro‐ecological zones: the mountains, the Senqu River Valley and the foothills. Sheep in the lowlands have evolved differently in their habitat than in other zones. Increased investment in the lowlands for meat production could be helpful as a secondary feature. The PC factor analysis method investigated the interdependence of the initial 12 body measures. These three primary components typically describe an animal′s overall size, meat qualities and form. The study found that PCs can be effectively utilised to select animals based on a set of linked variables. This is a critical feature for the conservation of animal genetic resources. The findings will be useful in future improvement initiatives for Merino sheep in Lesotho. Optimising sheep qualities through intelligent breeding tactics can result in higher meat and wool production, which benefits producers economically.

## Conflicts of Interest

The authors declare no conflicts of interest.

## Author Contributions

M.G.—designed the experiment, participated in the data collection and analysis, and drafted and critically revised the manuscript. M.J.M—drafted and critically revised the manuscript. O.W.O—designed the experiment, supervised the data collection and analysis and critically revised the manuscript. P.M‐R.—supervision of the data collection and revision of the manuscript. S.M.—revision and validation of the manuscript. M.K.—designed the experiment, participated in the data collection and analysis and revised the manuscript.

## Funding

This study was supported by the Wool and Mohair Promotion Project (Grant ID: 2000000818, 2000000818).

## Data Availability

The data that support the findings of this study are available from the authors upon reasonable request.
